# Matthew 7:3—a response to Kingsland and Taiz (2024)

**DOI:** 10.1007/s00709-024-02002-4

**Published:** 2024-11-19

**Authors:** Peter V. Minorsky

**Affiliations:** Mercy University, Dobbs Ferry, NY 10522 USA

**Keywords:** Jagadis Chandra Bose, Daniel T. MacDougal, Plant electrophysiology, Racism

## Abstract

I present here a rebuttal to an article in this volume wherein Kingsland and Taiz (2024) cast aspersions about an article I have written concerning Sir Jagadis Chandra Bose (Minorsky PV, in Plant Signal Behav 16:1818030, 2021) a brilliant Bengali scientist who was a pioneer not only in physics (microwaves and semi-conductors), but also in elucidating the electrophysiological responses of plants to environmental stimuli. The charge of racism that I have levelled at Bose’s most powerful and well-connected botanical adversary in the 1920s, Daniel T. MacDougal, is irrefutable: MacDougal was a racist, his racism extended to South Asians, and he used racist epithets in referring to Bose. MacDougal offered no cogent arguments against Bose’s electrophysiological measurements but attacked Bose with the racist trope that South Asians were “mystics.” MacDougal wielded his political and editorial clout to publicize faulty research in opposition to Bose while ignoring a sizable body of contemporaneous evidence in support of Bose’s ideas. Unfortunately, given MacDougal’s stature as the General Secretary of the American Association for the Advancement of Science (AAAS) and the racist tenor of the time, many Western scientists were too ready to accept uncritically MacDougal’s proclamations that Bose was a fraud, an incompetent, and a “Hindoo” mystic. Bose was one of the greatest minds to ever contemplate plant function. It is high time that we, in the West, redress this historical wrong, and acknowledge Bose’s enormous and revolutionary contributions to plant physiology.

## Introduction

… why beholdest thou the mote that is in thy brother’s eye,

but considerest not the beam that is in thine own eye?

Matthew 7:3

In their article “Plant ‘Intelligence’ and the Misuse of Historical Sources as Evidence,” Kingsland and Taiz (2024) charge that members of the plant neurobiology community have distorted the historical record by presenting false quotations, taking quotations out of context, doctoring quotations, and offering interpretations of quotations unsupported by the original texts. Among the plant neurobiology papers targeted by Kingsland and Taiz (2024) is my article “American Racism and the Lost Legacy of Sir Jagadis Chandra Bose, the Father of Plant Neurobiology” (Minorsky 2021). Given the title of Kingsland and Taiz’s contribution, less attentive readers might infer by association that my scholarship regarding Bose is flawed and that I am guilty of misusing historical sources. Kingsland and Taiz provide no examples of my having used false quotations or quotations taken out of context, or of offering interpretations of quotations that are not clearly supported by the original texts. Rather, Kingsland and Taiz charge me with the sin of “confirmation bias,” that is, using historical sources to support foregone conclusions. As will be seen, Kingsland and Taiz are guilty of confirmation bias themselves, as well as many of the same academic transgressions that they level at other members of the plant neurobiology community.

## Bose was a victim of racism

My research into Bose began as a plant electrophysiologist seeking to understand why Bose went from being the most internationally celebrated plant physiologist of the 1920s to a scientific pariah in the 1930s (Minorsky 2021). In particular, I was interested in why Bose’s electrophysiological investigations, which were so consonant with my own findings (Minorsky & Spanswick 1989; Minorsky 1989), had come to be roundly rejected by the scientific community at large. I soon discovered that one of the most powerful and well-connected scientists in the United States in the 1920s, Daniel T. MacDougal, General Secretary of the American Association for the Advancement of Science (AAAS) and Director of the Desert Laboratory in Tucson, Arizona, had orchestrated, along with a cadre of associates, a propaganda campaign against Bose with the intention of obliterating Bose’s scientific reputation by accusations of technical incompetence, fraud and mysticism.

Having thus identified the cast of characters responsible for the destruction of Bose’s reputation, I turned my attention to their possible motives. Since the association of the East with mysticism was at that time, in the West, a common racist trope, I wondered whether Bose’s detractors in the United States may have been infected by the especially pernicious racism of the era: this was not a foregone conclusion on my part but a hypothesis. After several summers reading century-old print media as well as MacDougal’s archived correspondences, I uncovered a trove of evidence revealing not just MacDougal’s racism but the racism of virtually every member of MacDougal’s circle of associates. As the online magazine *The Paper Clip*, noted, “In his seminal paper titled “American Racism and the Lost Legacy of Sir J.C. Bose,” Peter V. Minorsky compellingly brings to our notice the instances where Bose’s contributions were undermined due to his Indian heritage, highlighting a possible case of racial discrimination. With meticulous research and captivating examples, Dr. Minorsky sheds light on a disheartening reality that warrants our attention and reflection” (https://thepaperclip.in/what-draws-sir-jagadish-bose-to-asteroid-city/). Additionally, I discuss, in light of modern knowledge, the falsity of MacDougal’s claims that Bose was a fraud, a technical incompetent, and a mystic (Minorsky 2021).

Despite the unequivocal evidence I present in Minorsky (2021) regarding the eugenicist and racist beliefs held by MacDougal and his associates, Kingsland and Taiz are not even willing to concede this point. Kingsland and Taiz (2024)  argue that my “…charges of racism on the part of particular American scientists are in part indirect and implicit.” This is true, but by simple logic, this means that my accusations of racism are also in part direct and explicit, as for example, when MacDougal wrote in a letter, “I am not so enthusiastic over conditions in New Mexico and southern Arizona as to wish to take on a third of a continent more of the same kind. We have got a major problem in the negro and a minor problem in the Mexican and other foreign races and to take over another big section of the same kind would be pretty bad when mixed in with our kind of politics” (D. T. MacDougal to W. T. Hornaday, 5 Dec. 1919, Arizona Historical Society, MS 0452, Box 15, Folder 9)*.* Any denial that this quotation is anything but incontrovertible evidence of MacDougal’s racism simply cannot be taken seriously. And why focus exclusively on the indirect and implicit evidence that I present and ignore the direct and explicit? By failing to acknowledge the strongest information that is contrary to their beliefs, Kingsland and Taiz are themselves guilty of confirmation bias.

Kingsland and Taiz (2024) continue, “Minorsky focuses mainly on Daniel T. MacDougal and a group of colleagues in MacDougal’s circle and argues that the possibility that these people were sympathetic to the eugenics movement in the 1920s means that they would necessarily harbor racist views.” This is a naïve argument. Although there were some eugenicists who were ableists rather than racists, the majority of eugenicists were both. I would encourage Kingsland and Taiz to familiarize themselves with some of the popular American eugenics books from the early twentieth century, such as Madison Grant’s *The Passing of the Great Race* (Grant 1916)*,* and Lothrop Stoddard’s *The Rising Tide of Color Against White World Supremacy* (Stoddard 1920), the theses of which can be gleaned from their titles.

Kingsland and Taiz (2024) state furthermore that Minorsky, “…also notes that in private correspondence, some of them, including MacDougal, used language that he considers derogatory.” It is not just my opinion: MacDougal did indeed use unequivocally racist language, specifically “Babu” and “Hindoo,” in referring to Bose. Although “Babu” is a term of respect or affection among South Asians, racist Westerners of a century ago used the term derisively, possibly because of it phonetic similarity to the English word “baboon.” (indeed, while reading century-old back issues of the Bengali magazine *The Modern Review*, I came across a popular joke in Bengal at the time: a Scottish soldier stops a Bengali native in the street and asks him, “Tell me, what precisely is the difference between a Baboo and a baboon?” The Bengali ripostes, “Just one letter, Sir, like the difference between a ‘Scot’ and a ‘sot!’”) One need not read the yellowed, crumbling pages of old Bengali magazines, however, to understand that “Babu” was a derogatory epithet widely used by racist English-speaking Westerners of the time: one can also pick up a dictionary (see https://www.merriam-webster.com/dictionary/babu). As for “Hindoo,” I must admit that when I first encountered this term, I assumed in my naïveté that it was just an alternative spelling of “Hindu.” I soon realized, however, that when “Hindoo” was used by early twentieth-century American writers, what followed was more often than not, racist rhetoric so vile that it is too offensive to quote. Indeed, an encyclopedic dictionary of American ethnic slurs, *The Color of Words*, identifies “Hindoo” as a variant spelling that may lend itself to derogatory use (Herbst 1997). To confirm this interpretation, I corresponded with Michael J. Altman, professor of religious history at the University of Alabama and author of *Heathen, Hindoo, Hindu: American Representations of India, 1721–1893* (Altman 2017). Professor Altman kindly responded, “…[Y]ou’re right that the term had racial and even racist connotations. During the 1920s, when Bose was working, there was both a larger anti-Asian sentiment and anti-immigrant sentiment in the United States and a more narrow “anti-Hindoo” movement on the West Coast. The influx of South Asian immigrants to the West Coast caused a backlash. Most of those immigrants weren’t Hindu, but Sikhs from the Punjab. Nevertheless, the idea of a “Hindoo invasion” took off in the press and even led to some violence and riots. All of this led to the United States v. Bhagat Singh Thind case in the Supreme Court in 1923 that ruled that “Hindoos” were not white and therefore not eligible for naturalized citizenship.” In short, “Babu” and “Hindoo” were the epithets commonly used by racist Americans in the 1920s in referring to South Asians. Thus, the evidence presented in Minorsky (2021) establishes beyond any reasonable doubt that MacDougal was a racist, that his racism extended to South Asians, and that he used racist epithets in referring to Bose.

## The beams in Kingsland and Taiz’s eyes

In addition to confirmation bias, Kingsland and Taiz (2024) commit many other scholarly transgressions, including several of the types that they accuse plant neurobiologists of having committed. For example, one of the sundry charges Kingsland and Taiz (2024) have levelled at other plant neurobiologists is “…offering interpretations of quotations unsupported by the original texts.” Kingsland and Taiz, however, are themselves guilty of this infraction. The impetus for Kingsland and Taiz’s transgression in this case was a 1908 *Scientific American* article in which MacDougal wrote in reference to *Mimosa pudica*, “In no instance does the activity of the plant involve choice or decision, or anything except the most generalized form of consciousness” (MacDougal 1908, 175)*.* MacDougal’s rejection of the possibility of plant choice or decision is agreeable to Kingsland and Taiz, but they seem uneasy with MacDougal’s mention of “plant consciousness.” Thus, they attempt to reconcile MacDougal’s views with their own by arguing that MacDougal did not really mean that plants were conscious. Kingsland and Taiz (2024) write, “We can take the MacDougal’s use of the term ‘consciousness’ to mean that he did not think that plants had the same kind of consciousness (or self-awareness) as animals: the ‘most generalized’ form of consciousness was only the ability to respond to stimuli, nothing more.” Kingsland and Taiz’s re-interpretation of MacDougal’s words are not supported by the facts. In an article in the *New York Times* in 1909 (28 Feb 1909, F8), MacDougal wrote, “If the proposal of Woodbridge be accepted, that the coupling up of two forms of perceptions gives rises to consciousness, then this faculty may be possessed by some plants of which the narcissus, or Chinese lily, is an example.” MacDougal then discussed Vöchting’s (1882) finding that the orientations assumed by narcissus blossoms depend both upon the force of gravity and the direction of illumination. Thus, MacDougal was fully aware that plants are not just responding to stimuli as Kingsland and Taiz assert, but that plants have the ability to integrate information provided by multiple inputs to effect an optimal response.

A third example of Kingsland and Taiz’s (2024) flawed scholarship concerns their attempt to disprove the straw argument that Bose was the sole source of research on the electrophysiology of plants in the early twentieth century. Towards this effort, they present not their own historical analysis but uncritically accept and incorrectly re-state arguments put forth nearly half a century ago by Galston and Slayman (1979) in reference to the controversy surrounding *The Secret Life of Plants*. (Since modern plant neurobiologists universally and loudly disavow the pseudoscience presented in *The Secret Life of Plants*, it is difficult to explain why Taiz keeps bringing up this discredited work in his attacks against the modern plant neurobiology movement unless his motives are propagandistic.)

Kingsland and Taiz (2024) write, “Arthur Galston and Clifford Slayman, in a review of the ensuing [*Secret Life of Plants*] controversy in 1979, noted that the book left the impression that Bose was the only person doing important work on electrophysiology in the early twentieth century… [Galston and Slayman] agreed that Bose deserved great credit for pointing out functional similarities between the electrical and mechanical responses of plants and animals. But they then discussed other lines of research in bioelectricity that were going on at that time, including the American school of W. J. V. Osterhout in botany, research by zoologist Elmer J. Lund at the University of Texas, anatomical studies by Harold S. Burr at Yale, and work by botanist Henrik Lundegaardh in Sweden. The field was developing independently of Bose both in botany and in zoology.” In this short passage, Kingsland and Taiz commit three scholarly transgressions. First, they alter Galston and Slayman’s precise words by adding “…in the early twentieth century…” This may seem trivial but, as will be argued, it is not. Second, Kingsland and Taiz’s exposition suddenly becomes very muddy here. Indeed, it is unclear from this passage whether it is Kingsland and Taiz’s (2024) or Galston and Slayman’s (1979) opinion that the field of electrophysiology was developing independently of Bose. In fact, Galston and Slayman state no such thing. Kingsland and Taiz attach their opinion anonymously at the end of a long paragraph devoted to summarizing Galston and Slayman’s positions. Third, the meticulous scholarship of Kingsland and Taiz (2024) has vanished. Why are there no dates or citations in reference to the works of Osterhout, Lund, Burr, and Lundegårdh? Why are Kingsland and Taiz relying on the conclusions of Galston and Slayman rather than reading themselves the original papers of these supposed contemporaries of Bose? Alas, by not doing so, Kingsland and Taiz have committed a huge blunder relating to historical chronology.

Bose’s scholarly output tailed off abruptly in the 1930s due to health complications stemming from diabetes and hypertension to which he eventually succumbed in 1937. To be clear, Bose edited several *Transactions of the Bose Research Institute* and wrote a handful of minor articles in the early 1930s, but his enormously insightful contributions relating to plant electrophysiology were effectively over by 1929 following his publication of four books in 4 years (Bose 1926, 1927, 1928, 1929). In toto, during the first three decades of the twentieth century, Bose presented his results from many hundreds, if not thousands, of experiments concerning the electrophysiological responses of higher plants to environmental stimuli and wrote many books on the subject.

Now, let us examine the chronology and the subject matter of the electrophysiological contributions of the four scholars cited by Galston and Slayman (1979). Were Osterhout, Lund, Burr, and Lundegårdh, in fact, studying the electrophysiological responses of higher plants contemporaneously with Bose and with the same objectives as Bose during the first three decades of the twentieth century? Not at all. Lundegårdh employed electrodes not to investigate the responses of higher plants to environmental stimuli but rather to elucidate the mechanisms underlying ion transport in plants (Lundegårdh 1940). Burr’s interest in plants mostly centered around continuous, long-term measurements of standing potentials in trees (Burr 1942; 1945; 1947), but his insights into plant bioelectricity were all published in the 1940s well after Bose’s death. Similarly, Lund’s contributions to bioelectricity just barely overlapped with Bose’s active career (Lund and Kenyon 1927; Lund and Bush 1930; Lund 1931a, b; Lund 1932), and like Burr, the research of Lund and his team focused much less on the excitable responses of higher plants than on the continuous direct currents (“currents of rest”) that can be measured in living organisms in an unstimulated state. (It should also be noted that far from being received with open arms by the scientific community, as might be inferred from Kingsland and Taiz’s historical renderings, researchers of continuous direct currents at rest were also treated as scientific pariahs by the orthodoxy (Becker and Selden 1985).) Although Osterhout and his colleagues were among the first to measure action potentials (APs) in a characean alga (e.g., Blinks et al. 1929; Osterhout 1934), most of this work was published in the 1930s and 1940s, and much of it was of poor quality by modern standards. In their early contributions, their measurements employed a method in which electrical contact with the vacuole was obtained by killing a region of the cell with chloroform (e.g., Osterhout and Harris 1928). As pointed out by Findlay (1959), an obvious objection to this method is that the *Nitella* cells survived only for a few hours following such treatment and were probably in an abnormal condition during the experimental period. If Galston and Slayman, and for that matter, Kingsland and Taiz, had a better grasp of the history of plant electrophysiology, they might have more effectively cited the experiments published in *Protoplasma* by Umrath (1930), who inserted fine glass capillaries (filled with an electrical conducting medium) into *Nitella* cells and succeeding in measuring membrane potentials more in keeping with modern measurements (Bretag 2017). Finally, although the APs of characean algae (Umrath 1930) and embryophytes (Bose 1926) are similar in grand view, modern research underscores the fact that they are not identical and exhibit several differences at more granular levels of detail (Kisnieriene et al. 2022).

Kingsland and Taiz (2024) want their readers to believe that the four, early twentieth-century plant electrophysiologists mentioned by Galston and Slayman (1979) were working contemporaneously with Bose. In fact, these scientists were performing their electrophysiological studies not during the period from 1900–1930, but *after* Bose’s active career, during the period from 1930–1950. In an effort to downplay Bose’s originality, Kingsland and Taiz have tried to obfuscate this chronological distinction by categorizing Bose and the other four scientists as researchers “…in the early twentieth century.” Galston and Slayman (1979, 338) did not make this mistake: they wrote, “The other major component of the modern theory** began to emerge in the 1930s **[emphasis mine], from experiments carried out by [Lund, Burr, and Lundegårdh].”

Another scholarly transgression perpetrated by Kingsland and Taiz (2024) is their specious argument that “Minorsky cannot prove beyond doubt that the criticisms of Bose were mainly racially motivated, but he infers that this is the case.” Biographers, of course, cannot “prove” anything in a scientific sense. The historical reconstruction of a person’s life and career from archives and newspaper accounts is much like assembling a jigsaw puzzle purchased from a second-hand thrift shop: some pieces are invariably missing and one occasionally finds a piece that seems to belong to a different puzzle entirely. Another problem confronted by biographers is that historical figures, like all humans, evolve in their thinking over time and are sometimes not truthful to themselves or others. Biographers strive not for absolute proof, which is an impossible ideal, but rather for proof beyond a reasonable doubt. Proof beyond a reasonable doubt, of course, is insufficient to convince the unreasonable.

## On the repeatability of Bose’s experimental findings

A consequence of MacDougal’s campaign against Bose was that readers of his propaganda and later, some historians, were led to believe that all scientists had failed in their efforts to replicate Bose’s findings: this view is a fiction. Many aspects of Bose’s plant physiological research were confirmed or extended upon by his contemporaries (Dowling 1921; Kôketsu 1923; Stern 1924; Chodat and Guha 1926; Dixon and Bennet-Clark 1927, 1928; Molisch 1929a, b; Rao 1930). Even Bose’s report of electrical pulsations in stems found some tepid support. Cori (1931), based on her studies of plants using a D’Arsonval galvanometer (an instrument that Bose found to be less than ideal for studying plant pulsations), reported that she did occasionally record plant pulsations like those reported by Bose, but she found them to be more fleeting and irregular, and she doubted they were involved in the ascent of sap. The authors of these confirmatory papers, with the exception of Dixon and Bennet-Clark (1927, 1928), explicitly cited Bose as their inspiration. Thus, to address Kingsland and Taiz’s (2024) straw argument, Bose was not the *sole* person studying plant electrophysiology in the first third of the twentieth century. On the other hand, no other researcher of the time came even remotely close to matching Bose’s contributions toward understanding the electrophysiological responses of higher plants to environmental stimuli.

## Bose’s two theoretical blunders

Kingsland and Taiz (2024), in an attempt to belittle Bose’s legacy, focus on two theoretical blunders he made, the first relating to the hylozoistic idea that all matter is alive (Bose 1902); the second relating to the idea that active contractions are involved in the ascent of sap (Bose 1923). The point raised in this section is that Bose’s experimental findings, even those “supporting” this pair of theoretical blunders, have stood the test of time quite well.

Unlike Kingsland (1991), who dispassionately and amorally describes MacDougal’s Machiavellian scheming during an unrelated academic dispute in 1906, I am not guilty of hagiography by way of understatement. I state unequivocally that “Bose was not perfect: his overly speculative theories and overreliance on inductive reasoning, in some cases, made him a target for condemnation” (Minorsky 2021, 14). To use a baseball analogy, Bose was a “homerun hitter,” and those who “swing for the fences” often have a lower batting average and a higher frequency of strikeouts than other batters. Like imaginative scientists throughout history, Bose swung for the fences, and twice he struck out infamously. Bose is not unique in this respect. For example, René Descartes, Taiz’s philosophical guru, proposed that man’s rational soul was located in the pineal gland (Finger 1995). No one argues, however, that Descartes’ entire contribution to knowledge should be tossed aside because of this blunder.

Bose’s first “strikeout” occurred at the turn of the century when he noticed that a metallic coherer that he had developed for receiving radio waves became less sensitive under continuous use but returned to normal after a refractory period. Such “fatigue” reminded him of the properties of muscle. Further analogies between wires and the excitable tissues of living organisms led to a blurring in Bose’s mind of the boundary between so-called “non-living” metals and “living” organisms. His discoveries generated in his mind a Vedantic theme termed the “Boseian thesis” by Dasgupta (1999) that ran through all his subsequent research. Simply stated, the Boseian thesis was, “All is one.” Bose’s rush to present this fantastic, and with hindsight, incredibly naïve thesis was without doubt Bose’s greatest error, all the more so because his friends urged caution on his part. According to Mukherji (1983, 34), “[Sir Oliver] Lodge wrote to Bose:’Many congratulations on your very important and suggestive experiments but go slowly, establish point by point and restrain inspiration.’ [Lord] Rayleigh remarked: ‘going too fast! Proceed slowly.’” Besides excitement in his own mind about his grand hylozoic theory, there was another reason that Bose acted incautiously in presenting these ideas: his health was deteriorating rapidly. Soon after he presented the results of his wire experiments in Paris in 1901, he became alarmingly weak and pallid and went directly to England where he underwent surgery for unknown reasons. My speculation, and note that I have no medical credentials, is that Bose may have undergone an emergency splenectomy. Bose is known to have suffered from visceral leishmaniasis or *kala-azar *(“black fever”) that he reportedly contracted during a hunting trip to Assam as a young man. In its most serious form, *kala-azar* is potentially fatal if untreated. Other consequences, which can occur a few months to years after infection, include fever, damage to the spleen and liver, and anemia. At the beginning of the twentieth century, splenectomies were a common procedure for treating certain latent health effects relating to *kala-azar* (Rees et al. 1984). Even today, splenectomies are considered to be potentially dangerous operations.

To a modern reader, the Boseian thesis is preposterous. Metal wires are as useless for studying nervous conduction as are the *papier mâché* volcanoes made by children, for understanding vulcanology. As a graduate student, several of my initial attempts to read Bose’s works were aborted when I was confronted with the Boseian thesis. Indeed, that Bose was able to garner data in seeming support of this thesis caused me to question the very validity of his scientific apparatus. It turns out, however, that Bose was not guilty of poor lab technique in this instance. Guillaume (1908) reported that an oxidizable wire does show the “Bose effect” as soon as a thin layer of oxide forms: it is not observed when the wire is perfectly clean. Jean Perrin, later a Nobel Laureate, found that a silver or platinum wire may also be shown to exhibit the “Bose-Guillaume effect” by covering it with a thin, porous coating of non-conducting material, such as silver, oxide, kaolin, or gelatin. Perrin (1908) suggested that a thick electrical double layer forms inside the porous film, and that ions of one sign are squeezed out by the torsion, thus giving rise to a potential difference and a momentary current. More recently, Wei and Neuman (1970) suggested that electron tunneling may underlie the “nerve-like” activity of oxidized wires.

Whatever the electrochemical reasons, metallic wires (e.g., iron, tin, silver) immersed in strong acids (sulfuric or nitric acid) are capable of astonishingly nerve-like properties. From 1909 into the mid-1930s the American Ralph S. Lillie investigated these properties as a potential model for nervous conduction (Lillie 1918; 1920; 1928). His iron wires in nitric acid propagated electrical disturbances down their lengths, causing refractoriness and recovery in their wake; they had thresholds for initiating these traveling pulses; they could be excited or inhibited by electric currents; they exhibited accommodation and even rhythmic behavior (Lewis 1968). Thus, Bose’s experiments in this arcane field of electrochemistry seem to have been validated. Although Bose’s interpretation of his oxidized wire studies, from a historical perspective, strike one as ridiculous, from a contemporary perspective, they were much less so. After all, papers based on the “iron wire model” of nervous conduction were being published in prestigious journals such as *Science* (Lillie 1918), *Biological Reviews* (Lillie 1936) and especially *The Journal of General Physiology* (e.g., Bishop 1927). Plant physiologists in the 1920s, even if they were aware of Bose’s studies of oxidized wires decades earlier, had no reason to question his experiments in this area since the iron wire model of nervous conduction was still viable in the eyes of many reputable scientists. Kingsland and Taiz (2024) argument that Bose’s Western contemporaries were justified in rejecting Bose’s botanical insights because of his research decades earlier concerning the “excitability” of metal wires, is simply erroneous.

Bose’s second “strikeout” was his hypothesis that pulsating cells in the inner cortex of plants act peristaltically to drive the ascent of sap (Bose 1923). Bose hypothesized that there might be something akin to a “heart” in plants—not a multi-chambered heart like that found in mammals—but a more primitive structure like the peristaltic “aortic arches” found in earthworms. In order to ascertain the location of this hypothesized pulsating tissue, Bose advanced a fine-tipped extracellular electric probe by small increments into the stems and petioles of various plant species. He could detect no pulsatory activity at the epidermis but in each case as the probe reached a depth corresponding to the inner cortex, electrical oscillations were observed. Nearly a century after Bose’s discovery of “pulsating plant cells” in the inner cortex of plants, Choi et al. (2014) observed waves of calcium release propagating through the inner cortex of roots of *Arabidopsis thaliana* In all likelihood, Bose was studying electrical correlates of these Ca^2+^ waves. Moreover, it is important to emphasize that although Bose’s *theoretical interpretations* of his experimental results relating to the ascent of sap were erroneous, many of the *experimental results* that he presented in support of his ideas have subsequently found experimental support (Mozhaeva and Pil’shchikova 1978; Wessler et al. 2001; Zholkevich et al. 2003; 2005). In my view, it is entirely in the realm of the possible that electrical pulsations and Ca^2+^ waves in the inner cortex of plants may be involved in some sort of guttation-like process that produces a weak, unidirectional movement of water that is much too feeble to account for the ascent of sap.

In any case, vital theories of the ascent of sap have popped up repeatedly in the history of plant physiology, from Godlewski (1884) to Ursprung (1907) to Janse (1913) to Bose (1923) to Kundt (1998). Were Bose’s views concerning the ascent of sap so out of step with the times that his contemporaries were justified in dismissing his research as pseudoscience? The historically ascendant theory of the ascent of sap, the cohesion-tension hypothesis, was proposed in 1894 (Dixon and Joly 1894). There is often a delay of decades between the proposal of a new hypothesis and its near-universal acceptance. Indeed, Reinders (1910, 563), in discussing the cohesion-tension hypothesis and Godlewski’s “vital hypothesis” of sap ascent, remarked that “…opinion is still divided with regard to two hypotheses, the advocates of which combat the views of their respective opponents with remarkable asperity.” No doubt the popularity of vitalist theories of sap ascent decreased during the decades following Reinders (1910) assessment, but in the 1920s, vitalist theories were not completely moribund. Even as late as 1939, a paper was published in the *Annals of Botany* suggesting that the inhibitory effect of chilling on the ascent of sap was indicative of a role for living cells in the process (Handley 1939). So, again, Kingsland and Taiz (2024) are guilty of confusing modern versus contemprary perspectives.

**Fig. 1:**
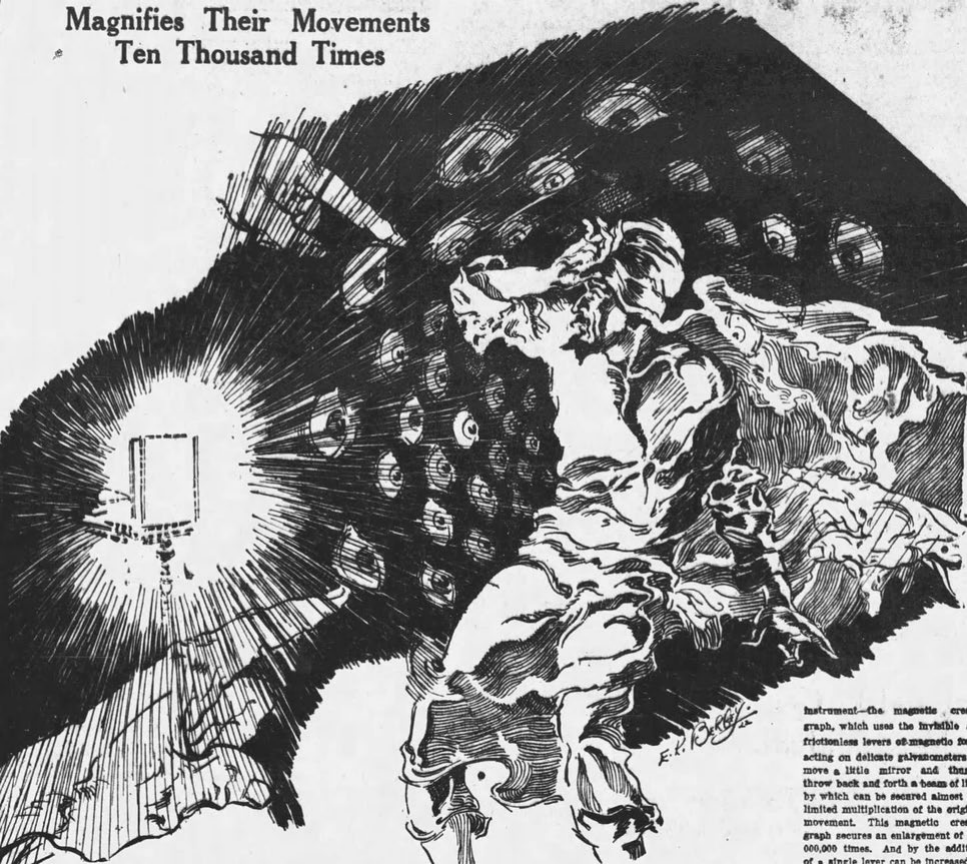
Bose in the thralls of scientific discovery as imagined by a 1930 American newspaper artist (with permission: *Wisconsin State Journal* (4 May 1930, page 32)

Reasonable people can admit that an individual might be correct about one topic (e.g., electrophysiological responses to environmental stimuli) and wrong about another (e.g., the ascent of sap). Many Western plant physiologists of the era, however, did not respond this way, largely because of MacDougal’s successful campaign against Bose. Living in a particularly racist era, many scientists were too ready to accept uncritically MacDougal’s proclamations that Bose was a fraud, an incompetent, a Hindoo mystic. MacDougal’s claim that Bose was a mystic was also promulgated in newspapers of the era (Fig. 1). Plant physiologists of the time came to dismiss Bose’s plant electrophysiological findings not for scientific reasons but because MacDougal wielded his prodigious editorial and political clout to promote only negative (and erroneous) objections to Bose’s findings (Minorsky 2021).

## Bose was more than just a scientist

Another flaw, in my opinion, in Kingsland and Taiz’s general approach to studying Bose is that they consider him solely as a scientist. When Bose delivered his Friday Evening Discourse at the Royal Institution in 1896, he was not just an ordinary scientist speaking for himself: he was a citizen of a colonized nation speaking as an equal before Europe’s most learned scientific scholars. Bose thus became a hero of all the colonized people of the world. At a time when many in the West considered Asians incapable of doing science, Bose’s public demonstrations of his research, which he performed literally around the world, were critical to raising the stature of all colonized people (Hoene 2018).

In addition to being a hero among colonized peoples, Bose was deeply committed to a modern branch of selfless, egalitarian, charitable, caste-free Hinduism (Brahmoism) that sought to share knowledge and creativity with *all* people. As a Brahmo, Bose rose to the challenge of educating people of every background and position, whether they be squatting field laborers, middle-class Western audiences, or members of the most learned societies. Mukherji (1983, 100) provides a marvelous example of Bose’s prowess as an educator of the scientifically illiterate. “In an inimitable style, Bose explains to the lay reader the phenomenon of the polarization of Hertzian waves. He asks the reader to imagine that a group of tortoises and storks is shooed in front of a vertical grating; only the storks can escape through it, but the tortoises cannot. If another vertical grating is placed before the storks after they have passed through the first grating, all of them can escape through the second. But if the second grating is turned through a right angle to make it horizontal to the ground, the storks cannot cross through this hurdle.” 

Bose’s lesson comparing polarized rays to storks and tortoises was not a preparatory lecture for entrance into graduate school but it did provide even the most uneducated a glimmer of insight into Bose’s discoveries in physics. As a public educator delivering a single lecture, one must take satisfaction in small increments in the understanding of the audience members, and not despair over the audience members’ lack of mastery.

Although Kingsland and Taiz, during their careers, gained success in the hallowed halls of Academia, neither to my knowledge has gained renown for their efforts in teaching the scientifically illiterate. One of the necessities of being a successful public educator in the sciences is to attract an audience. After all, if one lectures before an empty auditorium, one is not educating anyone. I can claim no experience as an educator of the scientifically illiterate, but I imagine that one key to success might be the simplification of scientific terminology. For example, one could give two identical lectures, the first entitled “Do Plants Feel Pain?,” the other entitled “Do Plants have Nociceptive Capabilities?” The first lecture with the more sensational, more familiar, and less accurate title might attract a healthy crowd, while the second lecture might attract only an audience of Lincoln Taiz. A second ploy to win over a crowd of the less intellectual and/or less educated is to indicate how the findings revealed relate to humans. For example*,* MacDougal who, to his credit, was also concerned about educating the general public, wrote in a popular article in the *New York Times* (28 Feb. 1909, p. F8), “Thus, a plant is sensitive to minute gradations of light not perceptible to the human eye, and likewise differences in contact of external bodies may be perceived by the plant far too delicate to be distinguished by the touch.” Kingsland and Taiz (2024), although they have never deigned to teach the general public, disparage those who use scientifically imprecise terminology and comparisons to humans in their efforts to educate the scientifically illiterate. In criticizing Bose, who was phenomenally successful in educating the masses, Kingsland and Taiz are reminiscent of unfit spectators in the bleachers shouting advice and admonitions to celebrated athletes. Although using imprecise terminology and falsely humanizing scientific content is unacceptable in an academic milieu, in the real world, they are, unfortunately, necessary for a greater good. It is simply unfair to apply the highest academic standards to the lectures of those struggling to teach the general public. It is also unfair to imply that individuals who take up the challenge of educating the masses are hucksters or fame-driven “publicity hounds.”

## Final thoughts

As we have seen, Kingsland and Taiz (2024) are guilty of some of the same academic sins that they decry in others: these include confirmation bias and offering interpretations of quotations unsupported by the original texts. Additionally, Kingsland and Taiz are guilty of presenting specious and straw arguments, of purposefully mischaracterizing statements made by previous scholars, of misrepresenting historical chronology, and of confusing modern versus contemporary perspectives.

In a recent paper, Clark et al. (2023) examined the social, psychological, and institutional causes and consequences of scientific censorship, which they define as actions aimed at obstructing particular scientific ideas from reaching an audience for reasons other than low scientific quality. Clark et al (2023, 3) add, “When scholars misattribute their rejection of disfavored conclusions to quality concerns that they do not consistently apply, bias and censorship are masquerading as scientific rejection.” Regrettably, “bias and censorship masquerading as scientific rejection” is, in my opinion, a perfect description of the unrelenting efforts of Taiz and other members of the “Spinach Inquisition” to derail the plant neurobiology revolution.

I began my historical investigation with the question of why Bose’s monumental and highly accurate experimental contributions concerning the electrophysiological responses of higher plants to environmental stimuli had come to be scorned. Other than erroneous accusations in the popular press that Bose’s electrophysiological investigations were artifacts of vibration, MacDougal never spelled out his objections to Bose’s electrophysiological investigations in a peer-reviewed forum (Minorsky 2021). Nonetheless, MacDougal (1932, 34), in his final commentary concerning the Bose affair, states, “The wholly mystic nervous mechanisms and pulsations of Bose have so far eluded all observation.” This was a lie in 1932, and despite Kingsland and Taiz’s depleted arguments, it remains a lie today.

## References

[CR1] Altman MJ (2017) Heathen, Hindoo, Hindu: American Representations of India, 1721–1893. Oxford University Press, New York

[CR2] Becker RO, Selden G (1985) The body electric: electromagnetism and the foundation of life. William Morrow, New York

[CR3] Bishop GH (1927) The effects of polarization upon the steel wire-nitric acid model of nerve activity. J Gen Physiol 11:159–174 19872388 10.1085/jgp.11.2.159 PMC2140964

[CR4] Blinks LR, Harris ES, Osterhout WJV (1929) Studies on stimulation in *Nitella*. Proc Soc Exp Biol Med 26:836–838

[CR5] Bose JC (1902) Response in the living and non-living. London, Longmans, Green

[CR7] Bose JC (1923) Physiology of the ascent of sap. London, Longmans, Green

[CR8] Bose JC (1926) The nervous mechanism of plants. London, Longmans, Green

[CR9] Bose JC (1927) Plant autographs and their revelations. MacMillan, New York

[CR10] Bose JC (1928) The motor mechanism of plants. London, Longmans, Green

[CR11] Bose JC (1929) Growth and tropic movements of plants. London, Longmans, Green

[CR12] Bretag AH (2017) The glass micropipette electrode: a history of its inventors and users to 1950. J Gen Physiol 149:417–430 28298356 10.1085/jgp.201611634 PMC5379916

[CR13] Burr HS (1942) Electrical correlates of growth in corn roots. Yale J Biol Med 14:581–588 21434041 PMC2601232

[CR14] Burr HS (1945) Diurnal potentials in the maple tree. Yale J Biol Med 17:727–735 21434237 PMC2601777

[CR15] Burr HS (1947) Tree potentials. Yale J Biol Med 19:311–318 20284283 PMC2602110

[CR16] Chodat RH, Guha SC (1926) La pollinisation et les réponses électriques du pistil. Arch Sci Phys Nat 8:105–111

[CR17] Choi W, Toyota M, Kim S, Hilleary R, Gilroy S (2014) Salt stress-induced Ca^2+^ waves are associated with rapid, long-distance root-to-shoot signaling in plants. Proc Nat Acad Sci USA 111:6497–6502 24706854 10.1073/pnas.1319955111 PMC4035928

[CR18] Clark CJ, Jussim L, Frey K, von Hippel W (2023) Prosocial motives underlie scientific censorship by scientists: a perspective and research agenda. Proc Nat Acad Sci USA 120:e2301642120 37983511 10.1073/pnas.2301642120 PMC10691350

[CR19] Cori M (1931) Cellule pulsanti e “correnti d’azione” nelle piante, in relazione all’ascensione dell’acqua. Annali di Botanica 19:465–482

[CR20] Dasgupta S (1999) Jagadis Chandra Bose and the Indian response to Western science. Oxford University Press, New York

[CR21] Dixon HH, Bennet-Clark TA (1927) Responses of plant tissues to electric currents. Sci Proc Roy Dublin Soc 18:351–372

[CR22] Dixon HH, Bennet-Clark TA (1928) Influence of temperature upon response to electrical stimulation. Sci Proc Roy Dublin Soc 19:27–38

[CR23] Dixon HH, Joly J (1894) On the ascent of sap. Ann Bot 8:468–470

[CR24] Dowling JJ (1921) Observations of plant growth with the recording ultramicrometer. Nature 107:523

[CR25] Findlay GP (1959) Studies of action potentials in the vacuole and cytoplasm of *Nitella*. Austral J Biol Sci 12:412–426

[CR26] Finger S (1995) Descartes and the pineal gland in animals: a frequent misinterpretation. J Hist Neurosci 4:166–182 11619024 10.1080/09647049509525637

[CR27] Galston AW, Slayman CL (1979) The not-so-secret life of plants: In which the historical and experimental myths about emotional communication between animal and vegetable are put to rest. Am Sci 67:337–344

[CR28] Godlewski E (1884) Zur Theorie der Wasserbewegung in den Pflanzen. Jahrb Wiss Bot 15:569–630

[CR29] Grant M (1916) The passing of the great race: or, the racial basis of European history. Scribner’s, New York

[CR30] Guillaume E** (**1908) Les Phénomènes de Bose et les Lois de l’Électrisation de Contact. Dissertation, Université de Zurich, Switzerland

[CR31] Handley WRC (1939) The effect of prolonged chilling on water movement and radial growth in trees. Ann Bot 3:803–813

[CR32] Herbst PH (1997) The color of words. An encyclopaedic dictionary of ethnic bias in the United States. Intercultural Press, Inc. Yarmouth, Maine, USA

[CR33] Hoene C (2018) Jagadis Chandra Bose and the politics of science. Gitanjali and Beyond 2:26–40

[CR34] Janse MJ (1913) Die Wirkung des Protoplasten in den Zellen welche bei der Wasserbewegung beteiligt sind. Jahrb Wiss Bot 52:603–621

[CR35] Kingsland SE (1991) The battling botanist: Daniel Trembly MacDougal, mutation theory, and the rise of experimental evolutionary biology in America 1900–1912. Isis 82:479–509

[CR36] Kingsland SE, Taiz L (2024) Plant ‘intelligence’ and the misuse of historical sources as evidence. Protoplasma (in press) 10.1007/s00709-024-01988-1 39276228

[CR37] Kisnieriene V, Trębacz K, Pupkis V, Koselski M, Lapeikaite I (2022) Evolution of long-distance signalling upon plant terrestrialization: comparison of action potentials in Characean algae and liverworts. Ann Bot 130:457–475 35913486 10.1093/aob/mcac098 PMC9510943

[CR38] Kôketsu R (1923) Über die Wirkungen der Elektrischen Reizung an den Pflanzlichen Zellgebilden. J Dept Agr Kyushu Imp Univ 1:1–133

[CR39] Kundt W (1998) The hearts of the plants. Curr Sci 75:98–102

[CR40] Lewis ER (1968) The iron wire model of the neuron: a review. In: Oestreicher HL, Moore DR (eds) Cybernetic Problems in Bionics (Bionics Symposium 1966). Gordon and Breach, New York

[CR41] Lillie RS (1918) Transmission of activation in passive metals as a model of the protoplasmic or nervous type of transmission. Science 48:51–60 17818906 10.1126/science.48.1229.51

[CR42] Lillie RS (1920) The recovery of transmissivity in passive iron wires as a model of recovery processes in irritable living systems: Part I. J Gen Physiol 3:107–128 19871842 10.1085/jgp.3.1.107 PMC2140407

[CR43] Lillie RS (1928) Analogies between physiological rhythms and the rhythmical reactions in inorganic systems. Science 67:593–598 17792499 10.1126/science.67.1746.593

[CR44] Lund EJ (1931a) External polarity potentials in the apex of the Douglas fir before and after mechanical stimulation. Plant Physiol 6:507–517 16652726 10.1104/pp.6.3.507 PMC440113

[CR45] Lund EJ (1931b) Electric correlation between living cells in cortex and wood in the Douglas fir. Plant Physiol 6:631–652 16652738 10.1104/pp.6.4.631 PMC440128

[CR46] Lund EJ (1932) Comparison of the effects of temperature on the radial and longitudinal electric polarities in wood and cortex of the Douglas fir. Plant Physiol 7:505–516 16652784 10.1104/pp.7.3.505 PMC440161

[CR47] Lund EJ, Bush M (1930) Electric correlation potentials in the leaf of *Bryophyllum*. Plant Physiol 5:491–509 16652677 10.1104/pp.5.4.491 PMC440239

[CR48] Lund EJ, Kenyon WA (1927) Relation between continuous bioelectric currents and cell respiration. 1. Electrical correlation potentials in growing root tips. J Exp Zool 48:333–357

[CR49] Lundegårdh H (1940) Salt absorption of plants. Nature 145:114–115

[CR50] MacDougal DT (1908) The faculties of plants. Sci Am 99(11):174–175

[CR51] MacDougal DT (1932) A half century of plant physiology. Ann Missouri Bot Gard 19:31–43

[CR52] Minorsky PV (1989) Temperature sensing by plants: a review and hypothesis. Plant Cell Env 12:119–135

[CR53] Minorsky PV (2021) American racism and the lost legacy of Sir Jagadis Chandra Bose, the father of plant neurobiology. Plant Signal Behav 16:1818030 33275072 10.1080/15592324.2020.1818030 PMC7781790

[CR54] Minorsky PV, Spanswick RM (1989) Electrophysiological evidence for a role for calcium in temperature sensing by roots of cucumber seedlings. Plant Cell Env 12:137–143

[CR55] Molisch H (1929a) The movement of sap in plants. Science 69:217–218 17789316 10.1126/science.69.1782.217-a

[CR56] Molisch H (1929b) Nervous impulse in *Mimosa pudica*. Nature 123:562–563

[CR57] Mozhaeva LV, Pil’shchikova NV (1978) Relationship between root pressure constituents and root water pumping rate. Dokl Akad Nauk SSSR 239:1005–1008 ([in Russian])

[CR58] Mukherji V (1983) Jagadis Chandra Bose. Ministry of Information and Broadcasting, Government of India, New Delhi, India

[CR59] Osterhout WJV (1934) Nature of the action current in Nitella: I. General considerations. J Gen Physiol 18:215 19872835 10.1085/jgp.18.2.215 PMC2141335

[CR60] Osterhout WJV, Harris ES (1928) Protoplasmic asymmetry in *Nitella* as shown by bioelectric measurements. J Gen Physiol 11:391–406 19872407 10.1085/jgp.11.4.391 PMC2140986

[CR61] Perrin J (1908) Le phénomène de Bose-Guillaume et l’électrisation de contact. C r Hebd Seanc Acad Sci, Paris 147:55–56

[CR62] Rao LN (1930) Physiological mechanisms in plants and animals. Half-Yearly J Mysore Univ 4:223–237

[CR63] Rees PH, Kager PA, Kyambi JM, Ayim EWN, Bhatt KM, Schattenkerk JK (1984) Splenectomy in Kala-Azar. Tropic Geograph Med 36:285–292 6506208

[CR64] Reinders E (1910) Sap-raising forces in living wood. Proc Royal Acad Sci, Amsterdam 12:563–573

[CR66] Stern K (1924) Elektrophysiologie der Pflanzen. Springer, Berlin, Germany

[CR67] Stoddard L (1920) The rising tide of color against white world-supremacy. Scribner’s, New York

[CR68] Umrath K (1930) Untersuchungen über Plasma und Plasmaströmung an Characeen. IV. Potential messungen an *Nitella mucronata* mit besonderer Berücksichtigung der Erregungserscheinung. Protoplasma 9:576–597

[CR69] Ursprung A (1907) Abtötungs- und Ringelungsversuche an einigen Holzpflanze. Jahrb Wiss Bot 44:287–349

[CR70] von Vöchting H. (1882) Die Bewegungen der Blüthen und Früchte*.* Bonn, M. Cohen & Sohn, Germany

[CR71] Wei LY, Neuman RH (1970) Conduction mechanisms in Lillie’s iron-wire model of nerve. Biophys J 10:818–833 5496904 10.1016/S0006-3495(70)86337-8 PMC1367816

[CR72] Wessler I, Kilbinger H, Bittinger F, Kirkpatrick CJ (2001) The non-neuronal cholinergic system: the biological role of non-neuronal acetylcholine in plants and animals. Jpn J Pharmacol 85:2–10 11243568 10.1254/jjp.85.2

[CR73] Zholkevich VN, Aniskin DN, Dustmamatov AH (2003) On the stimulatory effect of neuromediators on the root pumping effect. Dokl Biol Sci 392:138–141 10.1023/a:1026179905962 14650874

[CR74] Zholkevich VN, Emel’yanova IB, Suschenko SV (2005) Self-oscillations of water transport in the plant root. Dokl Biol Sci 403:269–271 16358569 10.1007/s10630-005-0108-8

